# Diphtheria

**Published:** 1871-01

**Authors:** D. J. McMillan

**Affiliations:** Biggsville, Ill.


### Correspondence.

### DIPHTHERIA.

BIGGSVILLE, ILL., *Dec. 10, 1870.*

PROF. DAVIS—*Dear Sir:* There has been an epidemic of Diphtheria in this place and surrounding country, and I thought perhaps you would like to hear something in regard to it. It commenced here Sept. 20, and has prevailed up to this present time. The first case that occurred, a little girl 7 or 8 years of age, was violent from the start. She was taken with chill and violent fever, and followed within an hour or two with dyspnœa, tonsils and fauces covered with the usual white exudation; pulse rather full and frequent. The fever gradually cooled down, and in two or three days was nearly gone; but the dyspnœa remained for near a week. This case, however, was not a fair specimen of what followed. The next two cases occurred soon after, and had but little fever from the commencement to the close. In fact, if it had not been for the dyspnœa that came on the second or third day, with the weak and frequent pulse, there would have been no seeming cause for alarm.

It was only after the dyspnœa had increased to an alarming extent, that my partner, Dr. McDill, was called—too late to do any good; the disease had already done its work. The youngest died, and the oldest, after remaining speechless for some time, gradually recovered. In the meantime, two children (twins) nursing at the breast, took it, one after the other, and both died.

The next case that proved fatal was the youngest child of my partner, a little girl not 2 years old. In this case, as in the second that took it, the disease came on so stealthily, that its march was scarcely manifest until croupy breathing indicated the false membrane was down on the vocal cords. In about 48 hours after this hoarse breathing, the child was a corpse, notwithstanding the ablest medical counsel from Monmouth was called in.

From this, cases occurred in various parts of the town, and gradually appeared here and there in the surrounding country. The farthest case in our practice, (and there has none occurred around us), was nearly six miles away from town. The cases happening in the country invariably got well. The epidemic influence, whatever that was, would seemingly lie dormant by spells, there being times from its commencement in September when there would be no cases on hand; and what is generally the case with other epidemics was not the case with this, *viz.*, other epidemics commenced with fatal cases and shaded off quite mildly towards the last; this assumed the malignant form almost from the outset, then gradually shaded off to the mild, and seemed to have died out, when week before last a fatal case occurred, and then another in quick succession.

At the present writing, there are no bad cases, and but few of any kind. The sufferers have been principally children, not more than 3 or 4 adults, so far as known, having taken it, and they had it in its lightest form.

As to the sequelæ, but little after-effect was produced. In one instance, a large abscess of one of the posterior cervical glands occurred. One case that proved fatal was complicated with purpura.

With but few exceptions, in those cases that occurred after the disease had reached its maximum, the patients speedily got well. One fact I took notice of was, that when the disease commenced abruptly, with a high grade of fever, even though there was slight dyspnœa connected with it, the patient invariably got well, while in most instances where the disease came on slowly, with but little fever, the white patches gradually extending, then hoarseness, the case would end fatally.

In regard to the treatment; while physicians in various parts of this country and Great Britain have occasionally grown enthusiastic over certain courses of treatment, we have nothing to add to the already superabundant number of remedies; neither are we at all enthusiastic over the results of different forms of treatment, for we have tried several.

When we were satisfied that the exudation had reached the lungs, and, perhaps, in some instances, only the trachea, we found with but few exceptions, that the different forms of treatment were unavailing.

The time-honored chlorate of potash, iron and quinine treatment seemed to do as well as any other; although in some instances where the patient could be got to inhale right, inhalations seemed to do good.

The inhaling material we generally used was after this formula:

℞ Iodine, gr. iv.

Idodide potassia, gr. iv.

Alcohol, ʒ iv.

Water, ℥ iv.

M.

A preparation of vinegar and sage was then made, *viz.*, a handful of sage was put into a pint of vinegar, which was brought to the boil in a vessel, and then put into a china or earthenware teapot, previously heated. A teaspoonful of the first preparation was then put into it, and the spout of the teapot was either placed at the child's mouth or nose, and the inhalation commenced. In the meantime, a lamp of some description was held under the teapot, to keep the fluid in it constantly boiling. The inhalations were repeated every few hours, and occupied, perhaps, on an average, ten minutes. We have never used inhalations alone, but only as an adjunct to other treatment, and only used it where there were symptoms of the trachea or lungs being affected. Where the child took it kindly, when there was dyspnœa, and where the preparation was rightly used, benefit seemed to follow. The great trouble, however, was to get the little patients to inhale; some could not be induced to inhale; and this is one reason why I write you this account. Is there any inhaling apparatus that can be used on infants and children in spite of their efforts to the contrary? The common apparatus with a mouth-piece for adults will not do much better than the teapot. If you know of any new apparatus that you could recommend, we would be much obliged for the information. It seems to us that medicated inhalations in bad cases where the membrane extends into the lungs, along with other constitutional treatment, is the rational way of treating the disease.

Any new method of treating this disease that you can give us would be thankfully received.

Yours, truly,

D. J. McMILLAN.

